# Placental mesenchymal stem cells suppress inflammation and promote M2-like macrophage polarization through the IL-10/STAT3/NLRP3 axis in acute lung injury

**DOI:** 10.3389/fimmu.2024.1422355

**Published:** 2024-11-15

**Authors:** Zhihao Nie, Qinglu Fan, Wanli Jiang, Shujian Wei, Renwei Luo, Haifeng Hu, Gaoli Liu, Yufei Lei, Songping Xie

**Affiliations:** ^1^ Department of Thoracic Surgery, Renmin Hospital of Wuhan University, Wuhan, China; ^2^ Institute of Life Sciences, Chongqing Medical University, Chongqing, China

**Keywords:** acute lung injury, NLRP3 inflammasome, macrophage polarization, placenta-derived mesenchymal stem cells, IL-10/STAT3 signaling

## Abstract

**Introduction:**

Acute lung injury (ALI) is a clinically severe respiratory disorder that currently lacks specific and effective pharmacotherapy. The imbalance of M1/M2 macrophage polarization is pivotal in the initiation and progression of ALI. Shifting macrophage polarization from the proinflammatory M1 phenotype to the anti-inflammatory M2 phenotype could be a potential therapeutic strategy. The intratracheal administration of placental mesenchymal stem cells (pMSCs) has emerged as a novel and effective treatment for ALI. This study aimed to investigate the role and downstream mechanisms of pMSCs in reprogramming macrophage polarization to exert anti-inflammatory effects in ALI.

**Methods:**

The study used lipopolysaccharide (LPS) to induce inflammation in both cell and rat models of ALI. Intratracheal administration of pMSCs was tested as a therapeutic intervention. An expression dataset for MSCs cultured with LPS-treated macrophages was collected from the Gene Expression Omnibus database to predict downstream regulatory mechanisms. Experimental validation was conducted through in vitro and in vivo assays to assess pMSCs effects on macrophage polarization and inflammation.

**Results:**

Both *in vitro* and *in vivo* experiments validated that pMSCs promoted M2 macrophage polarization and reduced the release of inflammatory factors. Further analyses revealed that pMSCs activated the signal transducer and activator of transcription (STAT)3 signaling pathway by secreting interleukin (IL)-10, leading to increased STAT3 phosphorylation and nuclear translocation. This activation inhibited NLRP3 inflammasome activation, promoting M2 macrophage polarization and suppressing the inflammatory response.

**Conclusion:**

The study concluded that pMSCs alleviated lung injury in an LPS-induced ALI model by inhibiting M1 macrophage polarization and proinflammatory factor secretion, while promoting M2 macrophage polarization. This effect was mediated via the IL-10/STAT3/NLRP3 axis, presenting a novel therapeutic pathway for ALI treatment.

## 1 Introduction

Acute lung injury (ALI) is a life-threatening respiratory disorder with high morbidity and mortality. ALI is characterized by diffuse alveolar damage and excessive pulmonary inflammation, resulting in diffuse interstitial and alveolar edema that eventually leads to refractory hypoxemia and progressive dyspnea (1, 2). Acute respiratory distress syndrome (ARDS) is considered the most severe form of ALI and the mortality rate is nearly 40% (3). Currently, there is no effective drug therapy for ALI, and only supportive care with protective mechanical ventilation is available (4). Therefore, it is necessary to investigate the pathogenesis of ALI/ARDS. A thorough understanding of the potential underlying mechanisms might contribute to the development of new effective therapeutic methods for ALI/ARDS.

Accumulating evidence suggests that mesenchymal stem cells (MSCs), which are multipotent stem cells isolated from various tissues such as bone marrow, adipose tissue, placenta, and umbilical cord, show potential for treatment of ALI because of their immunomodulatory and immunosuppressive effects (5–8). Recent evidence indicates that MSCs are an effective therapeutic strategy for ALI (9). The human placenta is enriched in large numbers of MSCs, and placental MSCs (pMSCs) have served as an alternative source of MSCs for experimental and clinical research (10). Our previous study has reported that pMSCs can ameliorate lipopolysaccharide (LPS)-induced inflammation (11). Other studies have revealed that pMSCs can alleviate ALI by reducing inflammation, protecting lung function, and modulating macrophage polarization (7, 11–13). However, the specific underlying molecular mechanism of the therapeutic effect of pMSCs remains to be elucidated.

There is increasing evidence of involvement of NLRP3 activation in the pathogenesis of ALI/ARDS (14, 15). A previous study demonstrated that umbilical cord-derived MSCs pretreated with heat shock inhibited NLRP3 inflammasome activation in macrophages during ALI (16). Another study indicated that bone-marrow-derived MSCs exerted therapeutic effects by inhibiting the Nrf2/HO-1/NLRP3 signaling pathway in ALI (17). However, whether pMSCs alleviate ALI by inhibiting NLRP3 inflammasomes remains unknown and deserves further investigation. Our study was based on LPS-induced acute lung inflammation in rats and a peritoneal macrophage inflammatory model *in vitro* to probe the specific mechanism of action of pMSCs on ALI, and to provide a theoretical basis for exploring new predictive biomarkers and novel therapeutic strategies.

## 2 Materials and methods

### 2.1 Reagents

LPS (#L8880) from *Escherichia coli* 05*5:B5* with ≥98% purity was purchased from Solarbio (Beijing, China). ATP lithium salt (#11140965001) and STAT3 inhibitor VIII, 5,15-DPP (#573109) were obtained from Sigma–Aldrich (St Louis, MO, USA). The selective NLRP3 inhibitor MCC950 (#HY-12815) was purchased from MCE (Shanghai, China). Animal-free recombinant human IL-10 (#HZ-1145) was obtained from Proteintech (Wuhan, China). IL-10Rα antibody (#sc-365374) and IL-10 antibody (#sc-365858) were from Santa Cruz Biotechnology (Santa Cruz, CA, USA). Ribobio (Guangzhou, China) provided siRNAs targeting IL-10 (si-IL-10) and the respective negative control siRNA (si-NC).

### 2.2 Isolation and primary culture of rat peritoneal macrophages

Peritoneal macrophages were isolated from rats as described previously (18). Primary peritoneal macrophages were prepared by intraperitoneal injection of 1 mL 4% thioglycollate (Solarbio). After 3 days, peritoneal exudate cells were collected by cold PBS (Solarbio). Macrophages were centrifuged and resuspended in primary macrophage culture system (ICell Bioscience Technology, Shanghai, China). The cells were maintained in a humidified incubator at 37°C with 5% CO_2_ and used for follow-up experiments.

### 2.3 Cell treatment and transfection

Rat pMSCs were purchased from ICell Bioscience Technology (RAT-iCell-e001) and cultured in a primary mesenchymal stem cell culture system. The cell characteristics are provided in **Supplementary Figure S1**. Cells were maintained at 37°C in a 5% CO_2_ humidified incubator. Peritoneal macrophages were seeded in six-well plates at a 10^6^ cells/well, followed by treatment with LPS at 1, 2 or 5 µg/mL for 0, 0.5, 1, 2, 4 or 24 h. To detect whether the LPS−induced peritoneal macrophage inflammatory model was established, tumor necrosis factor (TNF)-α (#E-EL-R2856) and IL-10 (#E-EL-R0016) in the cell supernatant were measured by ELISA (Elabscience Biotechnology, Wuhan, China). To detect the therapeutic effects of pMSCs in the macrophage inflammation model *in vitro*, Peritoneal macrophages were induced after 4 h priming with LPS at 5 µg/mL, and then by challenge with 5 mM ATP (Sigma-Aldrich) for 1 h. Peritoneal macrophages were cocultured with pMSCs (passage3-5) at 37°C for 48 h using Millicell Cell Culture Inserts (Sigma–Aldrich) before LPS/ATP stimulation. Macrophages were pretreated with MCC950 (1 μM) for 12 h to inhibit NLRP3 inflammasome signaling before LPS/ATP stimulation. IL-10Rα antibody (10 μg/mL) was used to block IL-10 functions and recombinant human IL-10 (100 ng/mL) was used to treated macrophages with LPS/ATP stimulation. 5,15-DPP (50 μM) was used to bind to and antagonize the function of STAT3. To knock down expression of IL-10 (IL-10^KD^ pMSCs), macrophages were incubated with IL-10 siRNA. Ribobio designed and synthesized the siRNAs targeting rat-IL-10 (si-IL-10) and the respective negative control siRNA (si-NC). Cell transfection was conducted using Lipofectamine 2000 (Invitrogen, CA, USA) according to the manufacturer’s instructions. siRNA sequences targeted IL-10 si-Il10 001 5′-GCCTTATCGGAAATGATCC-3′; si-Il10 004 5′-ACAGCCGGGAAGACAATAA-3′; and si-Il10 005 5′-GAGCAGGTGAAGAGTGATT-3′. To overexpress IL-10(IL-10^OE^ pMSCs), macrophages were transfected with lentivirus-IL-10 with a multiplicity of infection (MOI) of 200 or vehicle for 48 h.

### 2.4 Lipopolysaccharide-induced acute lung injury (ALI) rat model

All experimental procedures were approved and in compliance with the guidelines of the Laboratory Animal Ethics Committee of Clinical Research, Renmin Hospital of Wuhan University (WDRY2018-K048). A total of 20 male Sprague–Dawley rats (6 weeks old; 220–250 g body weight) were purchased from Hunan SJA Laboratory Animal Co. Ltd. The rats were maintained in specific pathogen-free conditions. The animals were randomly divided into four groups (1): control group, injected with 0.5 mL sterile saline; (2) LPS-induced ALI group, intravenously injected via the tail vein with 7.5 mg/kg LPS dissolved in 0.5 mL sterile saline solution (19); (3) LPS+pMSCs group(passage3-5), rats were anesthetized with 2% pentobarbital (30 mg/kg), and 10^5^ pMSCs were administered by intratracheal instillation 1 h after injection of LPS; and (4) LPS+MCC950 group, MCC950 (50 mg/kg) was intravenously injected 1 h before injection of LPS. After 3 days, the rats were euthanized and bronchoalveolar lavage fluid (BALF) and lung tissue specimens were collected as described previously (20). There were five rats in each group.

### 2.5 Analysis of BALF

To collect BALF, fresh lungs of the rats were lavaged with 1.0 mL ice-cold PBS (pH 7.3) three times. BALF was centrifuged at 600 × g for 5 min at 4°C. Total white blood cells in BALF were stained with Giemsa (Beyotime, Shanghai, China) and double-blind counted using a hemocytometer. The protein levels in the BALF supernatant were measured using a BCA Protein Assay kit (Beyotime).

### 2.6 Lung histopathology and lung injury scoring

Lung tissues were fixed in 10% formalin solution, embedded in paraffin wax and cut into 5-μm sections. To evaluate histopathological changes in lung tissue, the sections were stained with hematoxylin and eosin (H&E). Alveolitis, infiltration or aggregation of inflammatory cells in the airspace and alveolar hemorrhage were observed under a light microscopy (Nikon, Tokyo, Japan). Lung injury score was measured as described previously (21). The assessment criteria were: 0, normal; 1, <20% lung section damage; 2, 20%–50% lung section damage; 3, >50% lung section damage. The mean score was used for comparison between groups.

### 2.7 Cytokines ELISA experiments

The levels of inflammatory factors TNF-α, IL-1β,IL-18 and IL-10 in the cell supernatant and BALF were quantified by ELISA, including Rat TNF-α (Tumor Necrosis Factor Alpha) ELISA Kit (#E-EL-R2856); Rat IL-10 (Interleukin 10) ELISA Kit(#E-EL-R0016); Rat IL-1β (Interleukin 1 Beta) ELISA Kit (#E-EL-R0012); and Rat IL-18 (Interleukin 18) ELISA Kit(#E-EL-R0567) (all from Elabscience Biotechnology).

### 2.8 Flow cytometry analysis

Single cells suspensions were isolated from BALF in the ALI animal model and LPS−induced peritoneal macrophage inflammatory model. Cells were stained with Fc Block antibody (#D34-485, BD Biosciences, San Jose, CA, USA) to block the Fc receptors. The following antibodies were used for surface staining: PE-conjugated anti-mouse CD40 (clone157506); APC-conjugated anti-mouse CD54 (clone 116120); PE-conjugated anti-mouse CD163 (clone 156704); and APC-conjugated anti-mouse CD206 (clone 141708) (BioLegend, San Diego, CA, USA). The cells were acquired on a NovoCyte Flow Cytometer (Agilent Technologies, Santa Clara, CA, USA) and generated data was further analyzed using NovoExpress 1.6.2 software. The gating strategy is detailed in **Supplementary Figure S2**.

### 2.9 Western blotting

Total protein samples were extracted from tissues and cells using RIPA lysis buffer (Beyotime) with phenylmethylsulfonyl fluoride (Biosharp, Anhui, China) and phosphatase inhibitors (Beyotime). To separate the nuclear and cytoplasmic proteins of macrophages, the nuclear and cytoplasmic protein extraction kit (Beyotime) was used to obtain the cell lysate. The protein concentrations were determined by BCA protein assay kit (Beyotime). Equal volumes of the protein were separated by 10–15% SDS-PAGE and transferred to 0.45-μm polyvinylidene fluoride membranes (Millipore, San Diego, CA, USA). Unspecific protein binding sites were blocked by 5% skimmed milk (skim milk-TBST containing 0.1% Tween 20, 1:20 w/v) for 1 h at room temperature. Membranes were incubated with primary antibodies at 4°C on a shaker overnight, involving anti-NLRP3 (#ab263899), anti-ASC (#ab175449) and anti-GAPDH (#ab8245) (Abcam, Cambridge, MA, USA). Anti-Caspase1 (#22915-1-AP) and anti-Histone H3 (#17168-1-AP) were obtained from Proteintech (Wuhan, China). Anti-cleaved-caspase-1(#89332), Anti-STAT3(# 9139S) and anti-p-STAT3(Tyr705, # 9145S) were purchased from Cell Signaling Technology (Danvers, MA, USA), followed by incubation with anti-mouse-HRP (Proteintech, #SA00001-1) and anti-Rabbit-HRP (Proteintech, #SA00001-2). The bands were visualized using ECL reagent (Biosharp) and analyzed by ImageJ software.

### 2.10 RT-PCR RNA extraction and quantitative real-time PCR

Total RNA was isolated by RNA extraction reagent (Servicebio, Wuhan, China) from lung tissues and cells. cDNA was synthesized by First Strand cDNA Synthesis Kit (Servicebio). Quantitative real-time PCR was performed with QuantStudio™ 1 Real-Time PCR System (Thermo Fisher Scientific, Waltham, MA, USA) using 2 × SYBR Green qPCR Master Mix (Servicebio). Transcript levels of mRNA were normalized to GAPDH gene and the relative RNA levels were analyzed using the 2^−ΔΔCt^ method. The primers used are shown in **Table 1**.

**Table 1:** Primer sequences used in RT-PCR.

ID	Sequence (5′–3′)
GAPDH-F	GCAGTGGCAAAGTGGAGATT
GAPDH-R	TCTCCATGGTGGTGAAGACA
ASC-F	GACAGTACCAGGCAGTTCGT
ASC-R	AGTCCTTGCAGGTCAGGTTC
Caspase-1-F	AGGCACGGGACCTATGTGAT
Caspase-1-R	AGGGCAAAACTTGAGGGTCC
IL-18-F	GACAGCCTGTGTTCGAGGATATG
IL-18-R	TGTTCTTACAGGAGAGGGTAGAC
NLRP3-F	TCACAACTCGCCCAAGGAGGAA
NLRP3-R	AAGAGACCACGGCAGAAGCTAG
IL-1β-F	CAAATCTCGCAGCAGCACATC
IL-1β-R	TGTCCTCATCCTGGAAGGTC

### 2.11 Bioinformatic analysis and visualization

Bioinformatic analysis was performed on the GSE198806 dataset (https://www.ncbi.nlm.nih.gov/gds/?term=) that was submitted by Cai et al. (22), which contained MSCs cultured alone, MSCs cultured with PBS-treated macrophages (MR group) and MSCs cultured with LPS-treated macrophages (MRL group), and was based on the GPL24247 Illumina NovaSeq 6000 System. The raw data were analyzed using R packages (pheatmap and ggplot2) of R statistical software (version 4.2.2; http://www.r-project.org/) to obtain a heat map and volcano plots. The annotation of genes includes Gene Ontology (GO) annotation (http://geneontology.org/), Kyoto Encyclopedia of Genes and Genomes (KEGG) pathway annotation (https://www.kegg.jp/). Visualization of gene set enrichment analysis (GSEA, http://www.broad.mit.edu/gsea/) were performed.

### 2.12 Statistical analysis

All data in the present study were analyzed using GraphPad Prism software v 9.5.1 and presented as mean ± standard deviation (SD). Student’s unpaired *t*-test was used to compare two groups. One-way analysis of variance followed by Tukey’s multiple comparison test was applied for multiple comparisons. P < 0.05 is considered statistically significant.

## 3 Results

### 3.1 pMSCs attenuate LPS−induced ALI by modulating NLRP3 inflammasome *in vivo*

**Figure 1:**
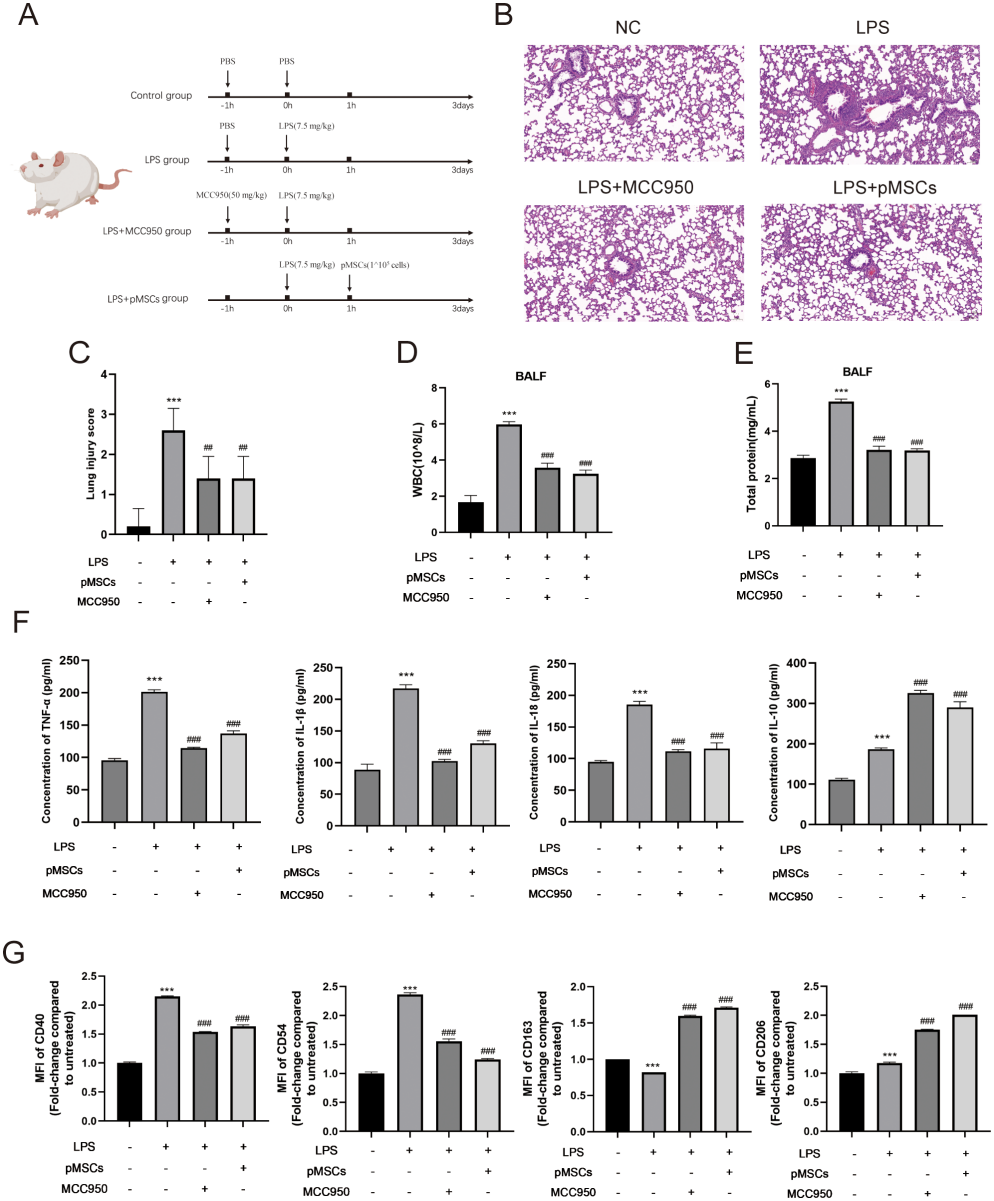
Intratracheal instillation of pMSCs alleviates inflammatory response in LPS-induced acute lung injury (ALI) rat model. (A) An outline of the design and schedule of the animal experiment. (B) Representative H&E staining images of lung tissue showing pathological changes. (C) lung injury score was used to examine LPS-induced histopathological changes. (Scale bar= 50 µm). (D) Inflammatory cells in bronchoalveolar lavage fluid (BALF) were counted. (E)Total protein concentration in BALF. (F) ELISA was conducted to determine levels of inflammatory cytokines of TNF-α, IL-1β, IL-18, and IL-10 in BALF. (G) Quantification of Median Fluorescence Intensity (MFI) of M1 (CD40 and CD54) and M2 (CD163 and CD206) macrophage polarization. Data are presented as the mean ± SD, n=5 per group. **P < 0.01 and ***P < 0.001 compared with PBS treatment. ^##^P < 0.01 and ^###^P < 0.001 compared with LPS at 7.5 mg/kg. Statistical significance was determined by student’s *t*‐test.

We explored the protective effect of pMSCs in the LPS-induced ALI animal model and the role of NLRP3 inflammasome in pMSCs protection. The rats were randomly divided into four groups: control, LPS, LPS+pMSCs, and LPS+MCC950. The experimental design is shown in **Figure 1A**. We examined the histopathological changes in lung tissues by H&E staining. Compared with the control group, the LPS group was characterized by diffuse alveolar injury with hyperemia and hemorrhage, inflammatory cell infiltration, interstitial edema and thickened alveolar septum, while pMSCs and MCC950 treatment of LPS-stimulated rats reduced lung tissue damage (**Figure 1B**). The lung injury scores in the LPS group were markedly higher than in the control group, whereas the scores in the LPS+pMSCs and LPS+MCC950 groups were decreased by about 40% compared with the LPS alone group (**Figure 1C**). Additionally, to evaluate the severity of alveolar capillary membrane injury, we measured the total leukocyte count and total protein concentration in BALF. After pMSCs or MCC950 treatment, the total cell count was significantly decreased compared with LPS treatment alone (**Figure 1D**). LPS stimulation markedly increased protein concentration in BALF, which was reduced by about 40% by LPS+pMSCs and LPS+MCC950 in comparison with LPS alone (**Figure 1E**). To establish the degree of inflammation in rat lungs in each group, we measured levels of inflammatory factors in BALF, such as IL-18, IL-1β, IL-10 and TNF−α. Compared with the control group, LPS treatment increased IL-18, IL-1β, IL-10 and TNF−α levels (**Figure 1F**). Compared with the LPS group, pMSCs and MCC950 markedly decreased TNF−α, IL-1β and IL-18 by about 32%/43%, 40%/53% and 38%/40%, respectively and increased IL-10 by about 35%/42% (**Figure 1F**). After pMSCs or MCC950 treatment, expression of M2 macrophage markers CD163 and CD206 was significantly increased, and expression of M1 macrophage markers CD40 and CD54 was significantly decreased (**Figure 1G**). Thus, pMSCs and MCC950 have a significant inhibitory effect on M1 polarization of macrophages. These data suggest that LPS induces ALI in rats, while pMSCs and NLRP3 inhibition can decrease ALI in rats induced by LPS.

**Figure 2:**
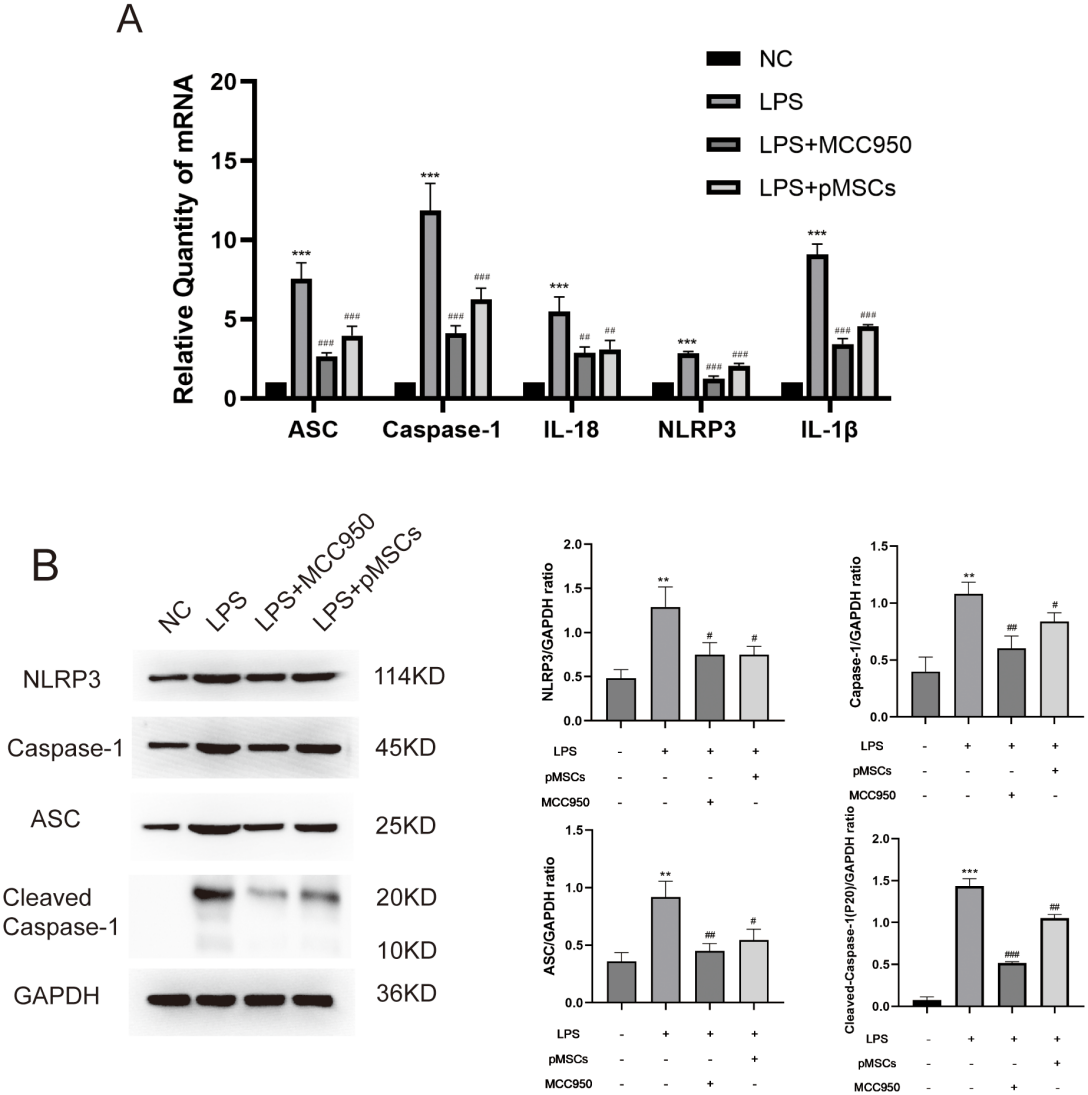
Intratracheal instillation of pMSCs downregulates NLRP3 inflammasome pathway expression in an LPS-induced acute lung injury (ALI) rat model. **(A)** mRNA levels of ASC, caspase-1, IL-18, NLRP3 and IL-1β were measured. Data are presented as the mean ± SD, n=5 per group. **(B)** Western blotting for NLRP3, caspase-1, ASC, Cleaved- caspase-1 and GAPDH in lung tissues. **P < 0.01 and ***P < 0.001 compared with PBS treatment. ^#^P < 0.05, ^##^P < 0.01 and ^###^P < 0.001 compared with LPS treatment at 7.5 mg/kg. Statistical significance was determined by Student’s *t*‐test.

The markers of NLRP3 inflammasomes, ASC (apoptosis-associated speck-like protein containing a CARD), caspase-1, cleaved-caspase-1, IL-18, NLRP3 and IL-1β, were detected in lung tissues. LPS stimulation significantly increased ASC, caspase-1, IL-18, NLRP3 and IL-1β mRNA and ASC, caspase-1, cleaved-caspase-1 and NLRP3 protein levels compared with the control group (**Figures 2A, B**). However, treatment with LPS+pMSCs and LPS+MCC950 decreased the NLRP3 inflammasome mRNA and protein levels in comparison with the LPS alone group. These data suggest that NLRP3 inflammasomes are involved in the protective effects of pMSCs against LPS-induced ALI in rats.

### 3.2 pMSCs inhibit NLRP3 inflammasome in LPS−induced macrophages inflammatory responses *in vitro*

**Figure 3:**
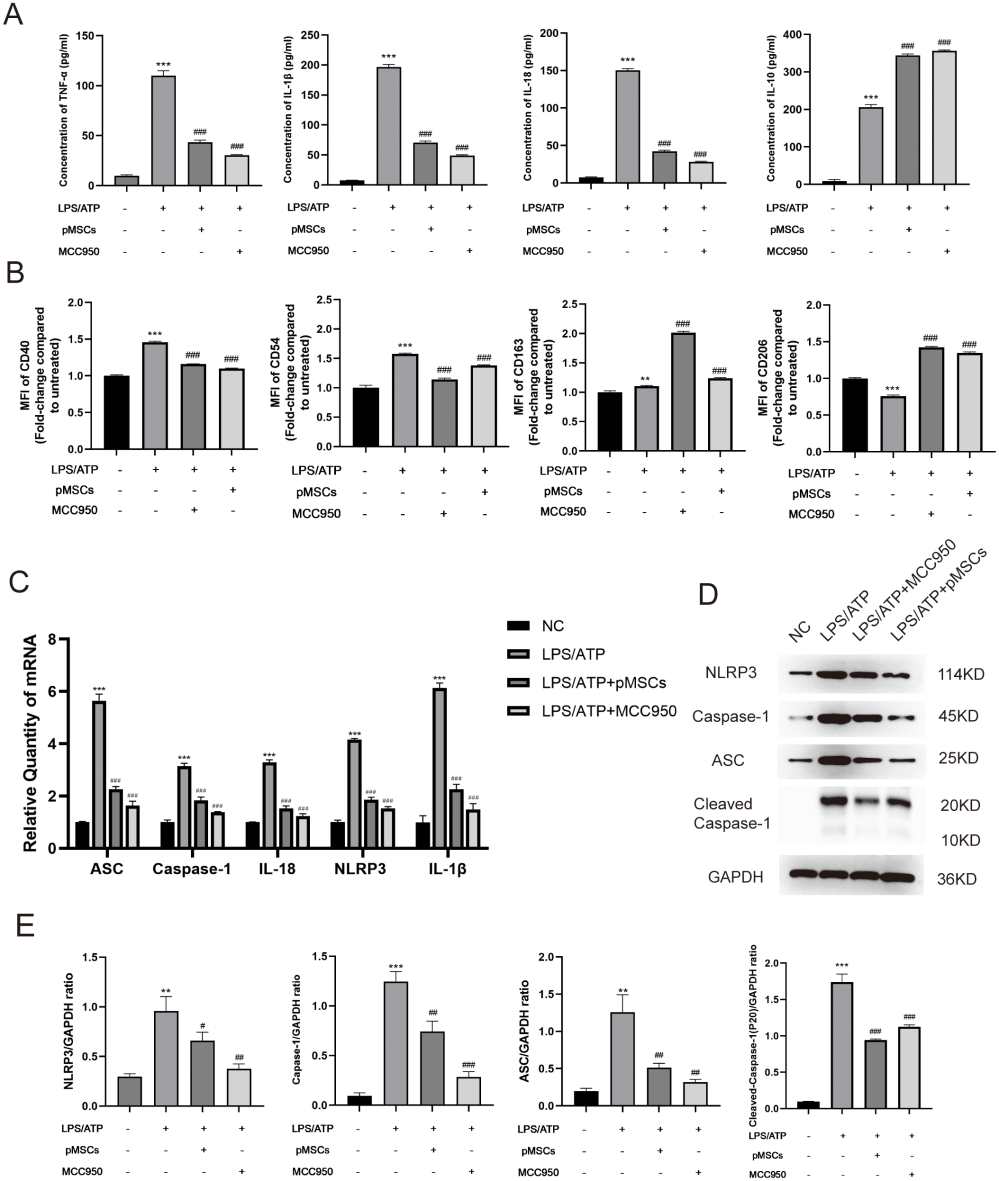
pMSCs attenuated inflammatory response in an LPS-induced peritoneal macrophage inflammation model via the NLRP3 inflammasome pathway. **(A)** Release of TNF-α, IL-1β, IL-18, and IL-10 was detected by ELISA. **(B)** Quantification of Median Fluorescence Intensity (MFI) of M1 (CD40 and CD54) and M2 (CD163 and CD206) macrophage polarization. **(C)** mRNA levels of ASC, caspase-1, IL-18, NLRP3 and IL-1β was measured. **(D, E)** Western blotting for NLRP3, caspase-1, ASC, Cleaved- caspase-1 and GAPDH. Data are presented as the mean ± SD, n=3 per group. **P < 0.01 and ***P < 0.001 compared with PBS treatment. ^##^P < 0.05, ^##^P < 0.01 and ^###^P < 0.001 compared with LPS pre-treatment at 5.0 μg/mL at 4 h plus ATP post-treatment at 5mM for 1h. Statistical significance was determined by Student's *t*‐test.

To establish an LPS−stimulated peritoneal macrophage inflammation model, the optimally induced concentration of LPS was assessed using TNF-α and IL-10 ELISA Kits. Four hours after LPS stimulation (5 µg/mL), the concentration of TNF-α and IL-10 was at peak level (**Supplementary Figure S3**). To explore the effect of pMSCs on LPS-induced ALI and the NLRP3 inflammasome, we divided macrophages into four groups: control, LPS+ATP, LPS+ATP+pMSCs, and LPS+ATP+MCC950. Cell viability and expression of cytokines, including TNF−α, IL-1β, IL-18 and IL-10, were detected. pMSCs and MCC950 treatment suppressed expression of IL-18, IL-1β and TNF−α and increased IL-10 in the cell supernatant compared with the LPS+ATP group (**Figure 3A**). After LPS/ATP stimulation, the expression of CD163 slightly increased, while CD206 expression significantly decreased. Following LPS+pMSCs and LPS+MCC950 treatment, CD206 expression markedly increased, and CD160 was slightly higher than in the LPS/ATP stimulation group, with statistical significance. After LPS/ATP stimulation, the expression of CD40 and CD54 significantly increased. Following treatment with LPS+pMSCs and LPS+MCC950, the expression of CD40 and CD54 significantly decreased (**Figure 3B**). NLRP3 inflammasome markers ASC, caspase-1, Cleaved- caspase-1, IL-18, NLRP3 and IL-1β were detected. pMSCs and MCC950 treatment decreased ASC, caspase-1, IL-18, NLRP3 and IL-1β mRNA levels and ASC, caspase-1, Cleaved- caspase-1 and NLRP3 protein levels compared with the LPS+ATP group (**Figures 3C–E**). These results show that pMSCs can inhibit LPS−induced inflammatory responses through negatively regulating expression of NLRP3 inflammasomes.

### 3.3 Bioinformatic analyses reveal MSCs upregulate the IL-10/STAT3 signaling pathway in LPS−induced macrophages inflammatory responses

**Figure 4:**
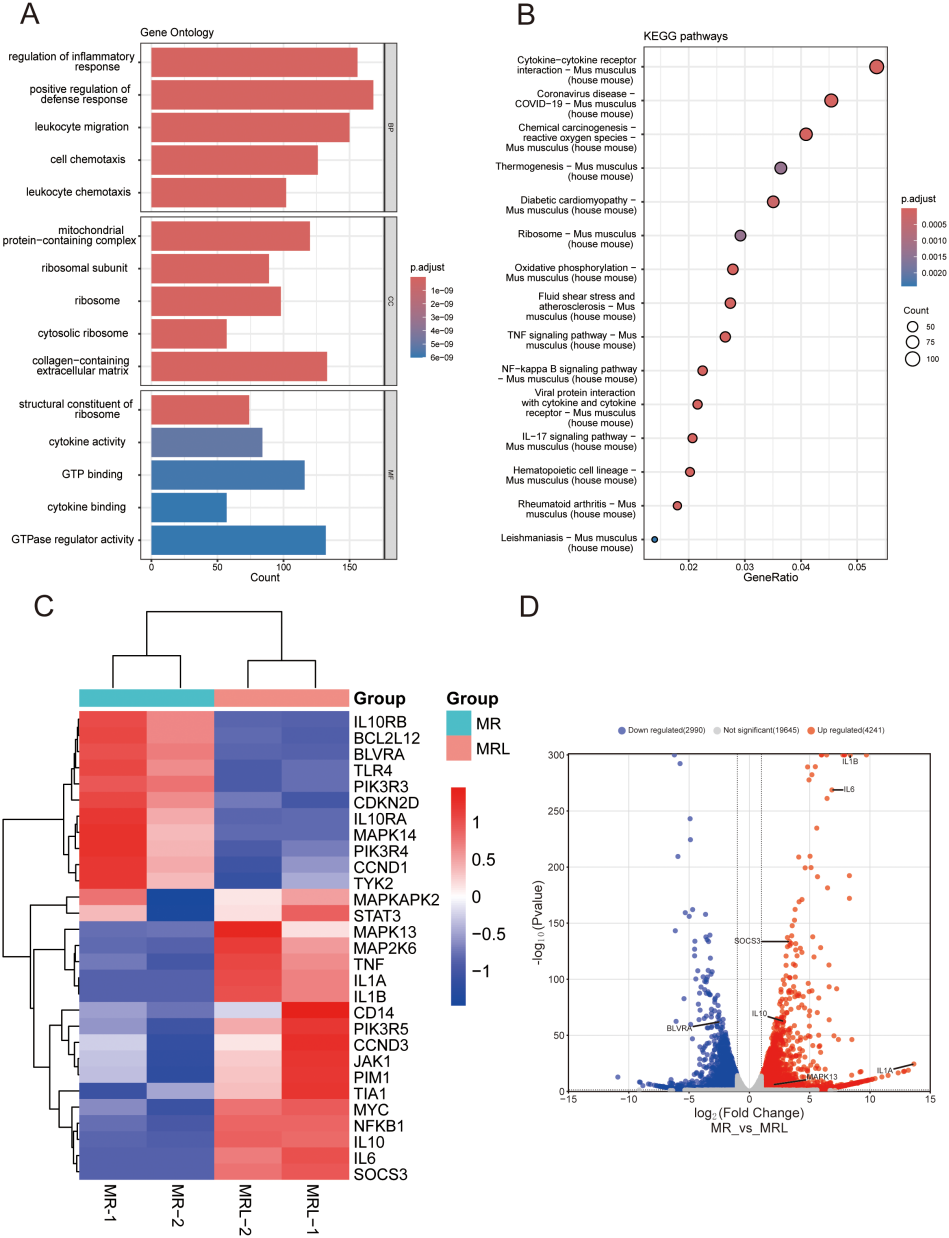
RNA-seq analysis of MSCs cultured with untreated or LPS-treated macrophages. **(A)** GO enrichment analysis: the top five biological processes, molecular functions, and cell components. **(B)** The top 15 KEGG enrichment analysis of upregulated differentially expressed genes. **(C)** Heatmap showing the core genes enriched in the IL-10-related pathway. The abscissa represents the sample number and the ordinate represents the differentially expressed genes: downregulated in blue, upregulated in red. **(D)** The volcano plot of differentially expressed genes and annotated with IL-10-related genes (|Log2(foldchange)| > 2, adjust P value < 0.05). downregulated in blue(N=2990), upregulated in red(N=4241).

Our previous study showed that pMSCs increased IL-10 during LPS-induced ALI (11). To explore the role of pMSCs in LPS-induced inflammation, we investigated anti-inflammatory and proinflammatory genes. Due to pMSCs express a profile consistent with MSCs, we downloaded datasets of MSCs cocultured with RAW cells (MR) or LPS-stimulated RAW cells (MRL) and analyzed the MSC transcriptome. Proinflammatory gene expression, including IL-6 and IL-1β, was downregulated in the LPS-induced macrophage plus MSCs group compared with the untreated macrophage plus MSCs and MSCs groups, while mRNA expression of anti-inflammatory gene IL-10 was significantly upregulated (**Supplementary Figure S4**). We then analyzed differentially expressed genes (DEGs) for the two groups (MR vs MRL). These genes were enriched by GO and KEGG analyses. GO analysis showed that the MRL group had changes in regulation of the inflammatory response and positive regulation of the defense response (**Figure 4A**). The MRL group was enriched in inflammatory and cytokine signaling pathways, such as the cytokine−cytokine receptor interaction pathway, TNF-α signaling pathway and NF-κB signaling pathway (**Figure 4B**). The DEGs were intersected with IL-10-pathway-related genes and IL-10/STAT3 was significantly upregulated in the MRL group (**Figures 4C, D**). According to the KEGG website (https://www.kegg.jp/pathway/map04630), IL-10 is located upstream of the JAK/STAT3 signaling pathway. Therefore, we hypothesized that pMSCs attenuate LPS-induced ALI by modulating the NLRP3 inflammasome via IL-10/STAT3 pathway.

### 3.4 pMSCs inhibit NLRP3 inflammasome through the IL-10/STAT3 pathway

**Figure 5:**
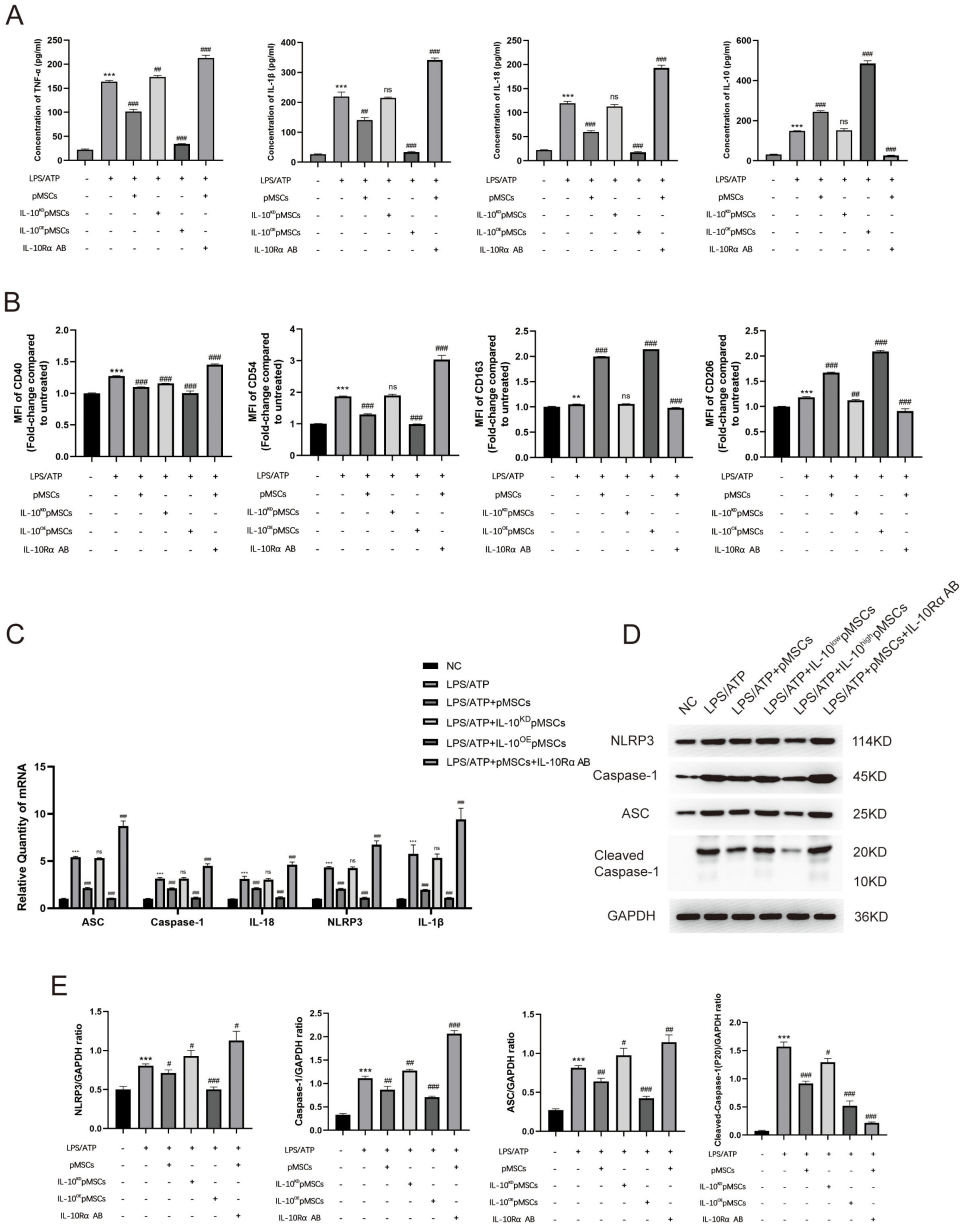
IL-10 inhibited the NLRP3 inflammasome pathway to prevent inflammatory response in an LPS-induced peritoneal macrophage inflammation model. **(A)** Release of TNF-α, IL-1β, IL-18 and IL-10 was detected by ELISA. **(B)** Quantification of Median Fluorescence Intensity (MFI) of M1 (CD40 and CD54) and M2 (CD163 and CD206) macrophage polarization. **(C)** mRNA levels of ASC, caspase-1, IL-18, NLRP3 and IL-1β was measured. **(D, E)** Western blotting for NLRP3, caspase-1, ASC, Cleaved- caspase-1 and GAPDH. This experiment was repeated at least three times with similar results. Data are presented as the mean ± SD, n=3 per group. **P < 0.01 and ***P < 0.001 compared with PBS treatment. ^#^P < 0.05, ^##^P < 0.01, ^###^P < 0.001 and ns indicates no significance, P > 0.05. compared with LPS pre-treatment at 5.0 μg/mL at 4 h plus ATP post-treatment at 5mM for 1h. Statistical significance was determined by student’s *t*‐test.

To investigate the underlying mechanism of action of IL-10 in LPS−induced inflammatory responses, we treated peritoneal macrophages in different ways. First, we investigated the expression of IL-10 in pMSCs cells (**Supplementary Figure S5**). Then, we divided macrophages into six groups: negative control; LPS+ATP; LPS+ATP+pMSCs; LPS+ATP+IL-10^KD^ pMSCs; LPS+ATP+IL-10^OE^ pMSCs; and LPS+ATP+pMSCs+ L-10Rα antibody. Expression of cytokines IL−10, IL-18, IL-1β and TNF-α was detected by ELISA. Cotreatment with pMSCs reduced expression of proinflammatory cytokines TNF-α, IL-1β and IL-18 and increased expression of anti-inflammatory cytokines IL-10 when compared with the LPS+ATP group (**Figure 5A**). Knockdown of IL-10 pMSCs cotreatment made no difference to expression of inflammatory cytokines compared with the LPS+ATP group. IL-10Rα antibody treatment further increased the expression of proinflammatory cytokines, while overexpression of IL-10 markedly decreased LPS-induced increases in expression of TNF-α, IL-1β and IL-18. Next, we detected the polarization of macrophages. Expression of CD163 and CD206 on macrophages was slightly increased after LPS/ATP stimulation, remained around markedly increased after LPS+pMSCs and this increased effect subsided after LPS+IL-10^KD^ pMSCs treatment. When used LPS+IL-10^OE^ pMSCs, the expression of CD163 and CD206 on macrophages was markedly increased and markedly decreased after treatment with LPS+pMSCs with added IL-10Rα antibody (**Figure 5B**). Expression of CD40 and CD54 on macrophages was significantly increased after LPS/ATP stimulation, after LPS+pMSCs, the expression of CD40 and CD54 markedly decreased and this decreased effect subsided after LPS+IL-10^KD^ pMSCs. After LPS+IL-10^OE^ pMSCs treatment, the expression of CD40 and CD54 on macrophages was decreased and markedly increased after treatment with LPS+pMSCs with added IL-10Rα antibody (**Figure 5B**).

We measured the levels of mRNA and proteins associated with NLRP3 inflammasomes. The LPS+ATP group showed higher expression of NLRP3-inflammasome-related mRNA (ASC, caspase-1, IL-18, NLRP3 and IL-1β) in comparison with the NC group (**Figure 5C**). Cotreatment with pMSCs, compared with LPS alone, decreased expression of NLRP3-inflammasome-related mRNA, which was further decreased by overexpression of IL-10 (**Figure 5C**). Knockdown of IL-10 pMSCs made no difference to the expression of NLRP3-inflammasome-related mRNA when compared with the LPS+ATP group (**Figure 5C**). The effects of IL-10 on NLRP3-inflammasome-related mRNA level were reversed by IL-10Rα antibody (**Figure 5C**). pMSCs inhibition of NLRP3 inflammasomes was also demonstrated by the protein levels of NLRP3, caspase-1 and ASC. The LPS+ATP group showed high expression of NLRP3-inflammasome-related proteins (NLRP3, caspase-1, Cleaved- caspase-1 and ASC), while the pMSCs+IL-10^OE^ pMSCs group showed an obvious reduction in expression of NLRP3-inflammasome-related protein (**Figures 5D, E**). The IL-10^KD^ pMSCs group and IL-10Rα antibody group further promoted NLRP3, caspase-1, Cleaved- caspase-1 and ASC protein levels in the cell inflammatory model.

### 3.5 IL-10 increases STAT3 activation and nuclear translocation in LPS−induced macrophages inflammatory responses

**Figure 6:**
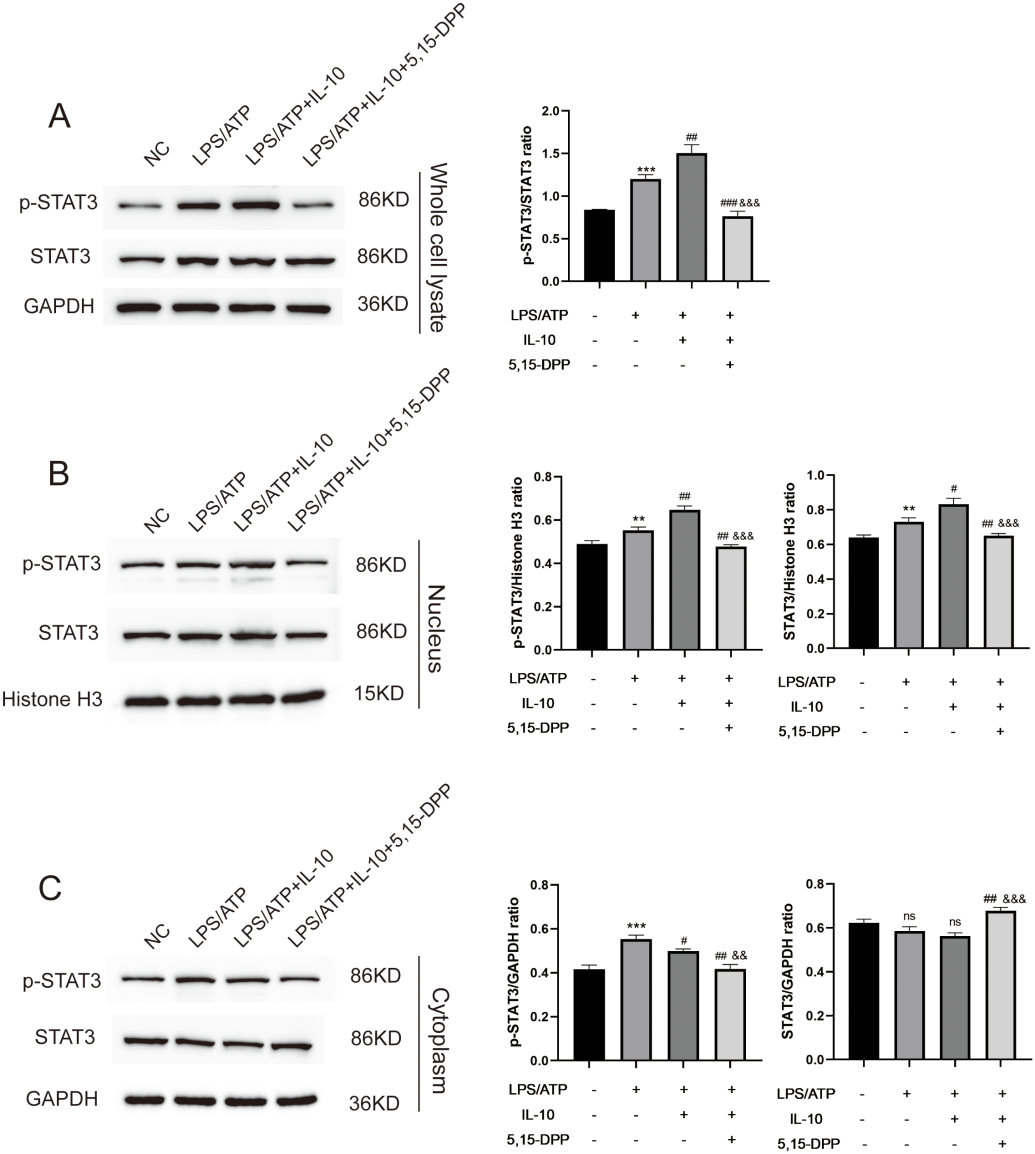
L-10 increased STAT3 nuclear translocation in LPS-induced peritoneal macrophage inflammation model. **(A)** Whole cell lysate detected expression of STAT3 and p-STAT3 Y705 in an LPS-induced peritoneal macrophage inflammation model after regulation of IL-10/STAT3 pathway. **(B, C)** Nuclear/cytoplasmic extraction and western blotting detected expression of STAT3 and p-STAT3 Y705.This experiment was repeated at least three times with similar results. **P < 0.01 and ***P < 0.001 compared with PBS treatment. ^*#*^P < 0.05, ^*##*^P < 0.01 and ^*###*^P < 0.001 compared with LPS pre-treatment at 5.0 μg/mL at 4 h plus ATP post-treatment at 5mM for 1h. ^*&&*^P < 0.01, ^*&&&*^P < 0.001 compared with LPS/ATP+IL-10 treatment. ns, no significant compared with Untreated and LPS/ATP sequentially. Statistical significance was determined by student’s *t*‐test.

The phosphorylation of STAT3 at Y705 leads to its dimerization, nuclear translocation, and DNA binding, which regulates gene transcription (23). To test whether IL-10 increased tyrosine phosphorylation of STAT3 in conjunction with its translocation from the cytoplasm to the nucleus in LPS−induced macrophages inflammatory responses, macrophages were divided into four groups: negative control; LPS+ATP; LPS+ATP+IL-10; and LPS+ATP+IL-10+STAT3 Inhibitor VIII (5,15-DPP). The initial analysis was conducted via Western blot using anti-STAT3 and anti-phospho-STAT3 (Tyr705) antibodies on whole-cell lysates. Compared to the untreated control, STAT3 expression was increased in the LPS/ATP group, the LPS/ATP+IL-10 group, and the STAT3 inhibition group. Similarly, p-STAT3 (Tyr705) was significantly upregulated in the LPS/ATP group and showed further enhancement upon co-treatment with IL-10, compared to the untreated control. The STAT3 inhibitor was able to significantly suppress the IL-10-induced expression of p-STAT3 (Tyr705) (**Figure 6A**). The cytoplasmic and nuclear extracts were subjected to Western blotting using anti-STAT3 and anti-phospho-STAT3 (Tyr705) antibodies. After IL-10 treatment, STAT3 levels detected in the nucleus increased, while the STAT3 signal in the cytoplasm slightly decreased at this time (**Figures 6B, C**). IL-10 treatment also enhanced the nuclear translocation of p-STAT3 (Tyr705). These results suggest that IL-10 increases STAT3 phosphorylation and induces translocation of p-STAT3 to the nucleus in LPS−induced macrophages inflammatory responses.

**Figure 7:**
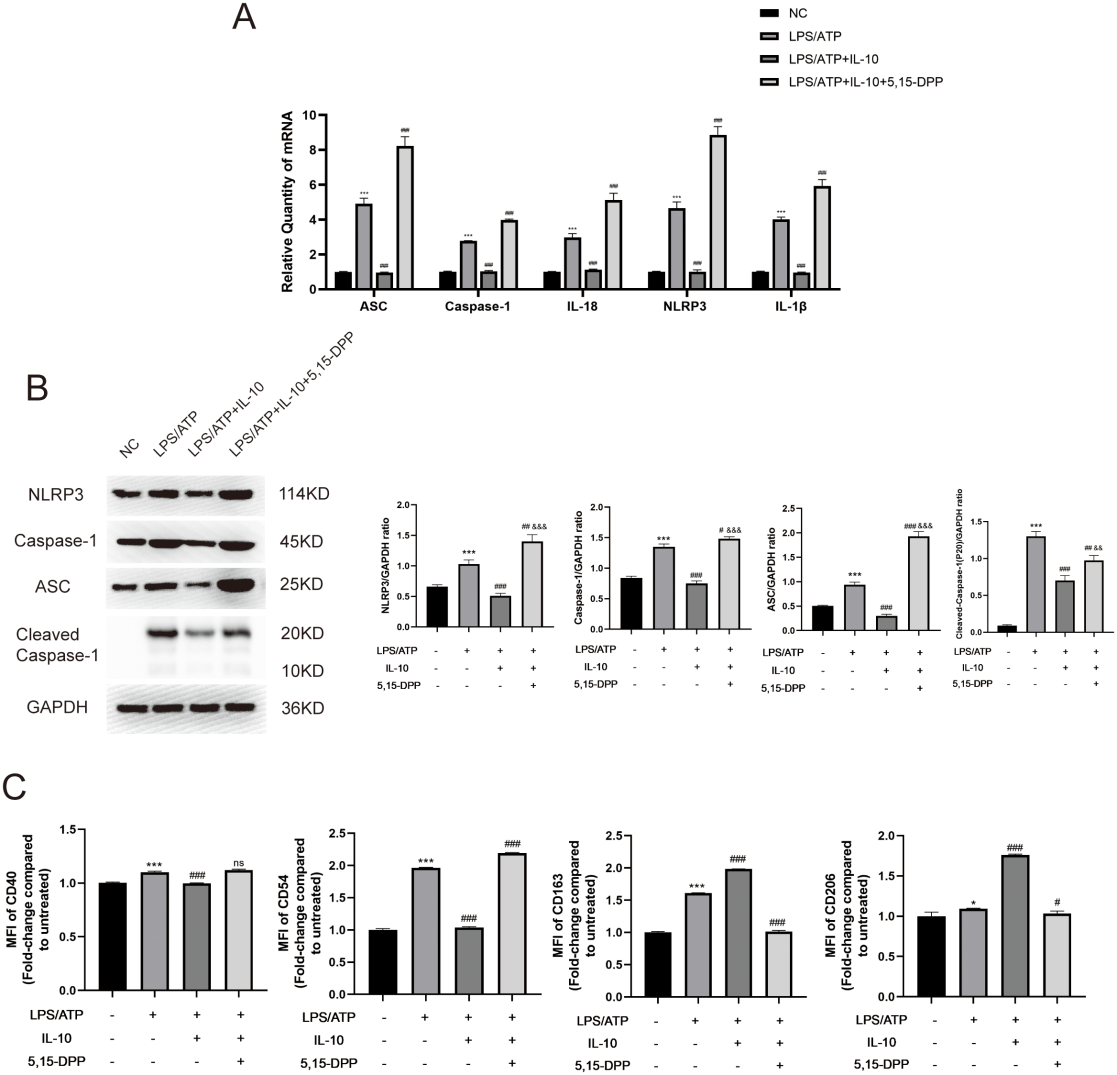
Inflammatory response was reduced in an LPS-induced peritoneal macrophage inflammation model via the IL-10/STAT3/NLRP3 signaling pathway. **(A)** mRNA levels of ASC, caspase-1, IL-18, NLRP3 and IL-1β were measured. **(B)** Western blotting for NLRP3, caspase-1, ASC, Cleaved- caspase-1 and GAPDH. This experiment was repeated at least three times with similar results. **(C)** Quantification of Median Fluorescence Intensity (MFI) of M1 (CD40 and CD54) and M2 (CD163 and CD206) macrophage polarization. Data are presented as the mean ± SD, n=3 per group. *P < 0.05, **P < 0.01 and ***P < 0.001 compared with PBS treatment. ^#^P < 0.05, ^##^P < 0.01 and ^###^P < 0.001 compared with LPS pre-treatment at 5.0 μg/mL at 4 h plus ATP post-treatment at 5mM for 1h. ^&&^P < 0.01 and ^&&&^P < 0.001 compared with LPS+IL-10 treatment. Statistical significance was determined by student’s *t*‐test.

For the JAK/STAT3 signaling pathway downstream of IL-10, we divided cells into four groups: negative control; LPS+ATP; LPS+ATP+IL-10; and LPS+ATP+IL-10+STAT3 Inhibitor VIII (5,15-DPP). LPS increased NLRP3-inflammasome-related mRNA levels (ASC, caspase-1, IL-18, NLRP3 and IL-1β). Cotreatment with IL-10 relieved the LPS-induced increase in NLRP3-inflammasome-related mRNA levels, while cotreatment with STAT3 Inhibitor VIII (5,15-DPP) further enhanced the LPS-induced increases (**Figure 7A**). Similarly, protein levels also showed that IL-10 significantly inhibited production of NLRP3-inflammasome-related proteins (NLRP3, caspase-1, Cleaved- caspase-1 and ASC) (**Figure 7B**). Inhibition of STAT3 significantly increased these proteins in the LPS-induced ALI cell model. When IL-10 was added to LPS-activated macrophages, expression of CD163 and CD206 on macrophages was significantly increased, but expression of CD40 and CD54 was significantly decreased. Conversely, the effect of M2 polarization of macrophages was blocked when STAT3 Inhibitor VIII (5,15-DPP) was administered to the LPS+ATP+IL-10 group (**Figure 7C**). These results indicate that pMSCs act against LPS-stimulated inflammatory responses by inhibition of the NLRP3 inflammasomes through the IL-10/STAT3 pathway *in vitro.*

## 4 Discussion

**Figure 8:**
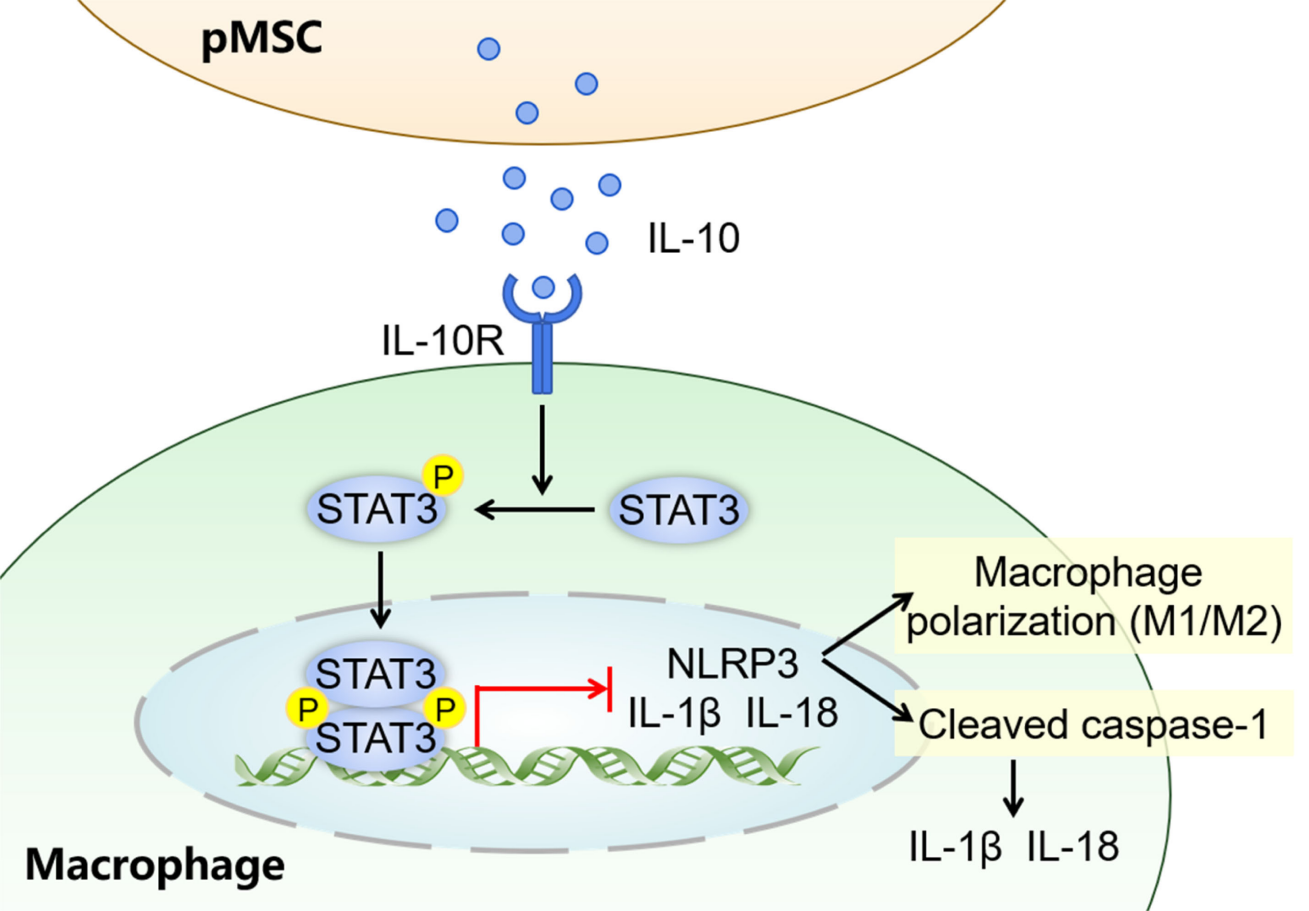
Graphical abstract for pMSCs alleviation of LPS-induced ALI. The possible mechanisms by which pMSCs activate downstream STAT3 signaling pathway by secreting IL-10 to alleviate LPS-induced ALI.

This study explored the potential therapeutic mechanisms of pMSCs for treating ALI. Our study confirms that pMSCs can alleviated LPS−induced ALI by activating the IL-10/STAT3 signaling to suppress NLRP3 inflammasome activation, as well as inhibiting M1 macrophage polarization and secretion of proinflammatory factors (**Figure 8**). The pathogenesis of ALI is complex and there are currently no clinically effective treatments (24).

Lung macrophages play a key role in the initiation and progression of inflammation in ALI/ARDS (25, 26). Macrophages can polarize into two distinct phenotypes, including classically activated M1 macrophages and alternatively activated M2 macrophages (27). In the early acute phase of ALI/ARDS, resident alveolar macrophages are activated and polarized from M2 to M1 with release various proinflammatory factors. In the later phase, activated and recruited M1 macrophages turn back to the M2 phenotype (25). Unlike M1 macrophages, M2 macrophages are involved in immunomodulation and tissue remodeling and repair (28). Therefore, remodeling the appropriate M1/M2 balance is a potential target for ALI/ARDS. Therefore, the pathogenesis of ALI must be studied to find more effective therapeutic targets. MSCs are multipotent somatic stem cells found in fat, bone marrow, umbilical cord, and many other locations. MSCs play a crucial role in immunomodulation, as well as self-renewal and differentiation (29). In recent years, several studies have revealed that MSCs play an important role in ameliorating ALI. MSCs protect lung tissue damage mainly by suppressing proinflammatory cytokines (TNF-α), producing anti-inflammatory cytokines (IL-1Rα and IL-10) and reducing extravasation of neutrophils into alveolar cavities (9, 30). As we reported previously (11), MSCs derived from human amniotic membranes provided protection in *in vivo* and *in vitro* models of lung injury. The results of the present study showed that the therapeutic effect of pMSCs in ALI therapy may be due to their unique immunoregulatory properties, including anti‐inflammatory cytokine (IL‐10) secretion and inhibition of proinflammatory cytokine (TNF-α, IL-1β and IL-18) expression, which is in agreement with previous findings (7). Importantly, MSCs regulate the polarization of macrophages (13). Abumaree et al. revealed a new immunosuppressive property of pMSCs, which converts macrophages from the M1 to M2 phenotype (31). In our study, administration of pMSCs reversed the elevated expression of inflammatory factors, but also increased the polarization of macrophages into M2 type (anti-inflammatory), which is consistent with the findings of previous studies performed by Morrison et al. (32). In recent years, multiple studies have illustrated that IL-10 is an effective anti-inflammatory cytokine, and increasing IL-10 production during ALI treatment can alleviate inflammation in injured lung tissues (33–35). Our previous study found that pMSCs can inhibit the inflammatory response by increasing the IL-10 secretion in an LPS-induced RAW264.7 macrophage inflammatory model (11). In this study, we used the GEO database to describe the potential mechanisms. Pathway analysis revealed upregulation of IL-10/STAT3 signaling in the MRL (MSCs+RAW264.7 macrophages+LPS) group. IL-10-mediated STAT3 has an anti-inflammatory effect. IL-10 binds to its receptor and the complex leads to activation of the downstream JAK/STAT3 signaling pathway. STAT3 activation (Tyr705) leads to STAT3 dimerization and nuclear translocation and induces the inhibition of the transcription and expression of proinflammatory genes (36). Some studies have found that peripheral-blood-derived and adipose-derived MSCs can regulate STAT3 signaling and promote M2 macrophage polarization by releasing IL-10 (37, 38). Therefore, to validate whether IL-10 secreted by pMSCs promotes the anti-inflammatory effect in ALI by activating STAT3, we used overexpression lentivirus and siRNA to interfere with the expression of IL-10, and IL-10R neutralizing antibody and STAT3 inhibitor (5,15-DPP) to regulate the IL-10/STAT3 signaling pathway. We found that activation of the anti-inflammatory IL-10/STAT3 signaling pathway suppressed inflammation by inhibiting M1 macrophage polarization and secretion of proinflammatory factors through blocking NLRP3 activation. Cao et al. and Liu et al. revealed that STAT3 phosphorylation activation and nuclear translocation attenuates LPS-induced ALI by inhibiting the activation of NLRP3 inflammasomes (39, 40), which agrees with our findings.

Macrophage polarization is crucial for immune homeostasis. Regulating the M1/M2 polarization status of macrophages can affect the severity of ALI (41). The polarization of macrophages from M1 to M2 phenotype is necessary for ameliorating pulmonary inflammation and mitigating injury during acute pulmonary infections (13, 26, 42). Wu et al. noted that the heme-catabolizing enzyme heme oxygenase 1 promotes macrophage polarization via the TXNIP/NLRP3 signaling pathway (43). Similarly, Bonda et al. confirmed that advanced glycation end products promote M1 macrophage polarization via the same pathway (44). Thus, we explored the relationship between NLRP3 signaling and macrophage polarization in the context of ALI. We found that MCC950 blocked the NLRP3 inflammasome, which inhibited M1 macrophage polarization while promoting M2 macrophage polarization. However, the specific mechanisms underlying macrophage polarization regulated by NLRP3 inflammasomes are not well understood. More in-depth research is required to verify the effect of NLRP3 inflammasomes on macrophage polarization.

There were some limitations to this study. Although we discovered that pMSCs exerted anti-inflammatory effects in ALI by secreting IL-10, it should not be ignored that M2 macrophages also secrete IL-10 (35). Further experiments are required to distinguish between the effects.

## 5 Conclusions

The present study verified that pMSCs inhibited LPS-induced ALI by inhibition of M1 macrophage polarization and secretion of proinflammatory factors, which was mediated via the novel IL-10/STAT3/NLRP3 axis. Our study provides data toward elucidating the molecular mechanism underlying regulation of the M1/M2 macrophage polarization balance by pMSCs in exerting anti-inflammatory effects. Despite its limitations, the above results provide a theoretical basis for new anti-inflammatory targets of pMSCs.

## Data Availability

The datasets presented in this study can be found in online repositories. The names of the repository/repositories and accession number(s) can be found in the article/**Supplementary Material**.
