# Supplementary material for: Placental mesenchymal stem cells suppress inflammation and promote M2-like macrophage polarization through the IL-10/STAT3/NLRP3 axis in acute lung injury
Source: Front Immunol. 2024 Nov 15;15:1422355. doi: 10.3389/fimmu.2024.1422355 (PMC11604576; doi:10.3389/fimmu.2024.1422355)
Supplement: Supplementary file 1 [file DataSheet1.docx]

Supplementary Material


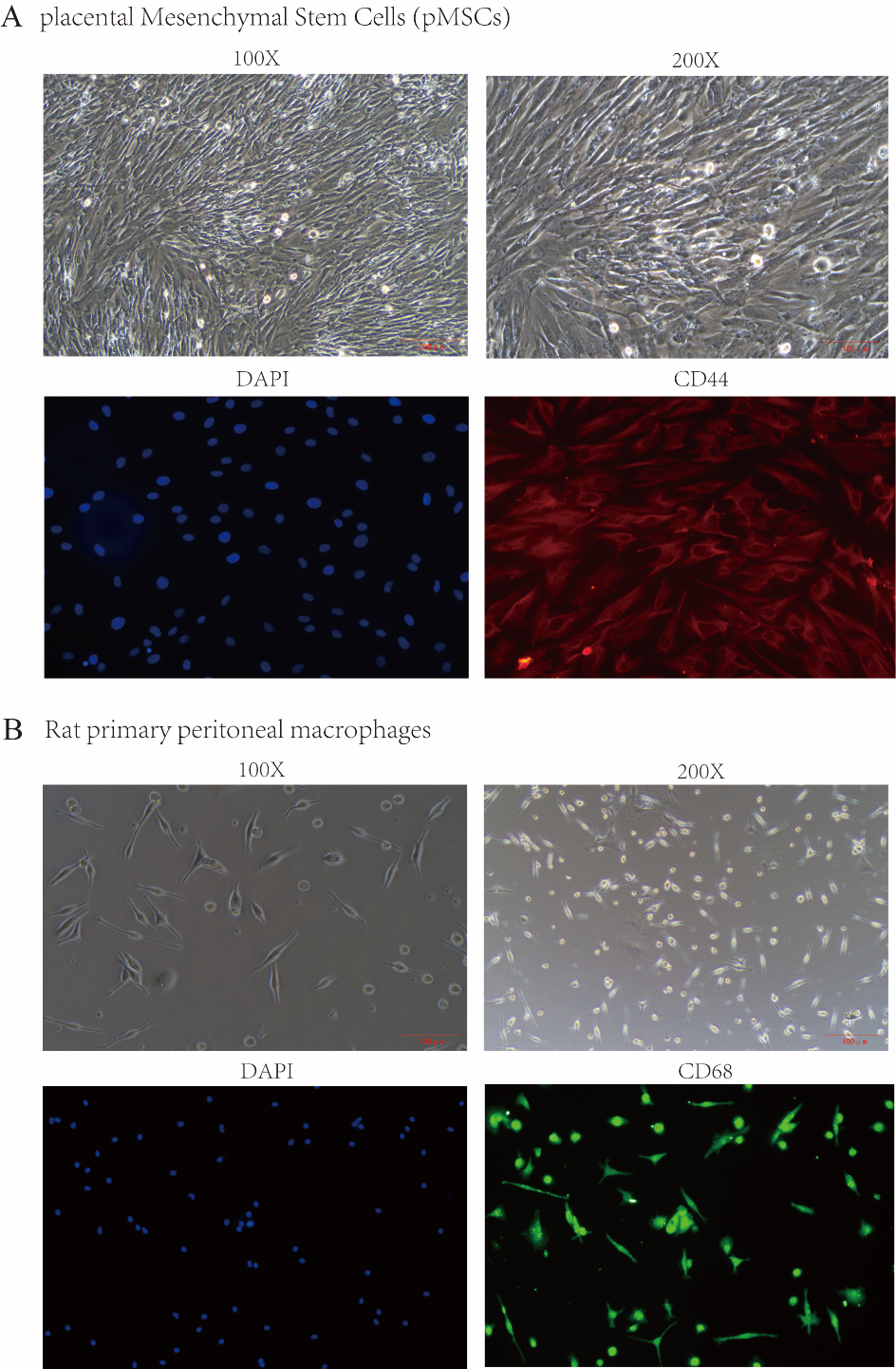


**Supplementary Figure S1.** Characterization of Placental Mesenchymal Stem Cells (pMSCs) and Rat Primary Peritoneal Macrophages. (A) Placental Mesenchymal Stem Cells (pMSCs): Representative images of pMSCs at 100X and 200X magnification (top row). The pMSCs used in experiments were at passage (P3-P5). Immunofluorescence staining shows DAPI-stained nuclei (blue) and CD44 expression (red) in pMSCs (bottom row). (B) Rat Primary Peritoneal Macrophages: Representative images of rat primary peritoneal macrophages at 100X and 200X magnification (top row). Immunofluorescence staining shows DAPI-stained nuclei (blue) and CD68 expression (green) in macrophages (bottom row).


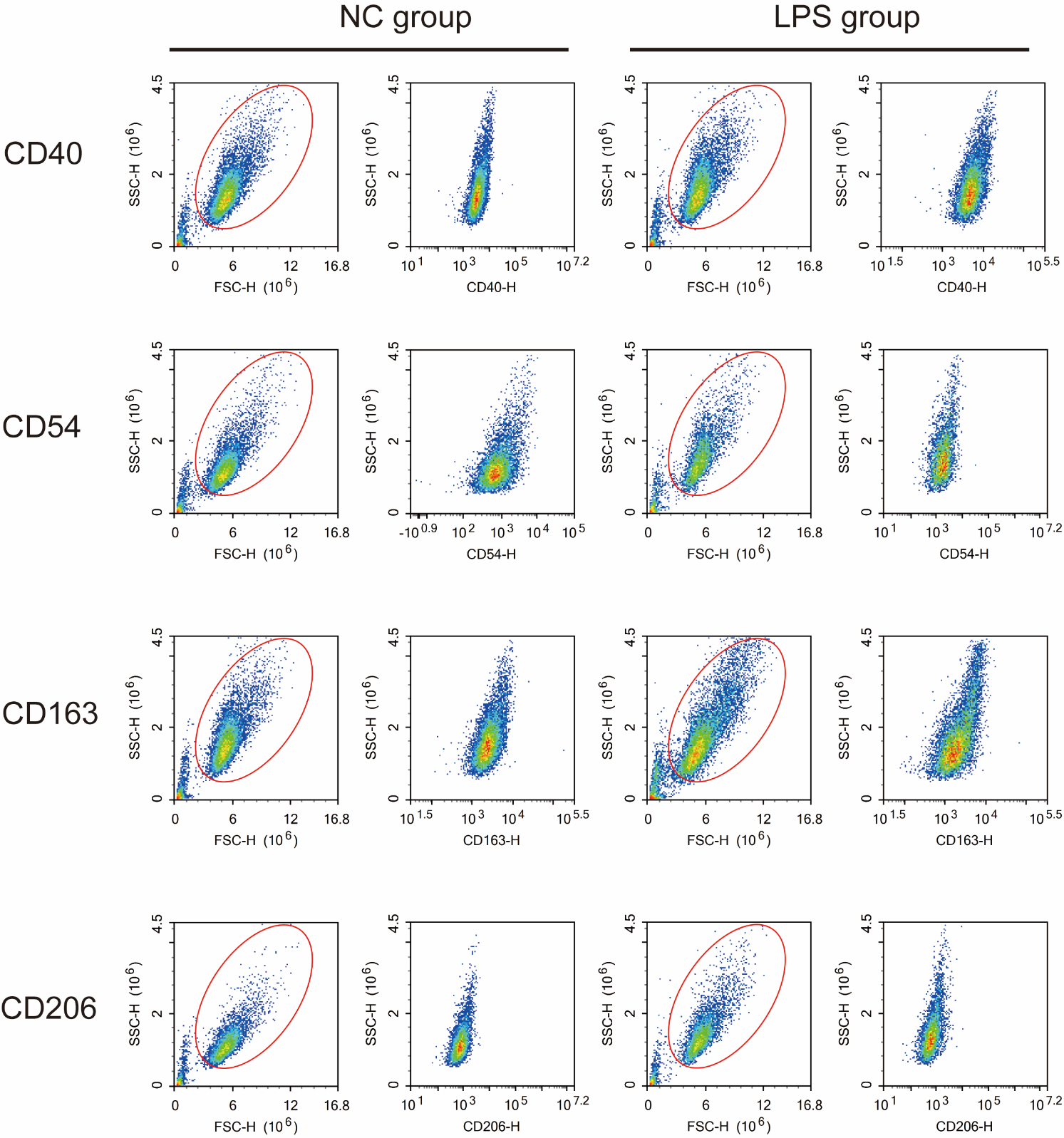


**Supplementary Figure S2.** **Gating Strategy for Flow Cytometry Analysis of CD40, CD54, CD163, and CD206 Expression.** Representative flow cytometry plots showing the gating strategy used to identify cell populations expressing CD40, CD54, CD163, and CD206（NC VS LPS group）. For each marker: Left Panel: Forward scatter (FSC-H) vs. side scatter (SSC-H) plot used to gate the cells of interest (red ellipse). Right Panel: SSC-H vs. CD marker fluorescence intensity plot used to identify the specific population expressing the marker of interest (CD40, CD54, CD163, or CD206). This gating strategy was applied to quantify the median fluorescence intensity (MFI) of each marker.


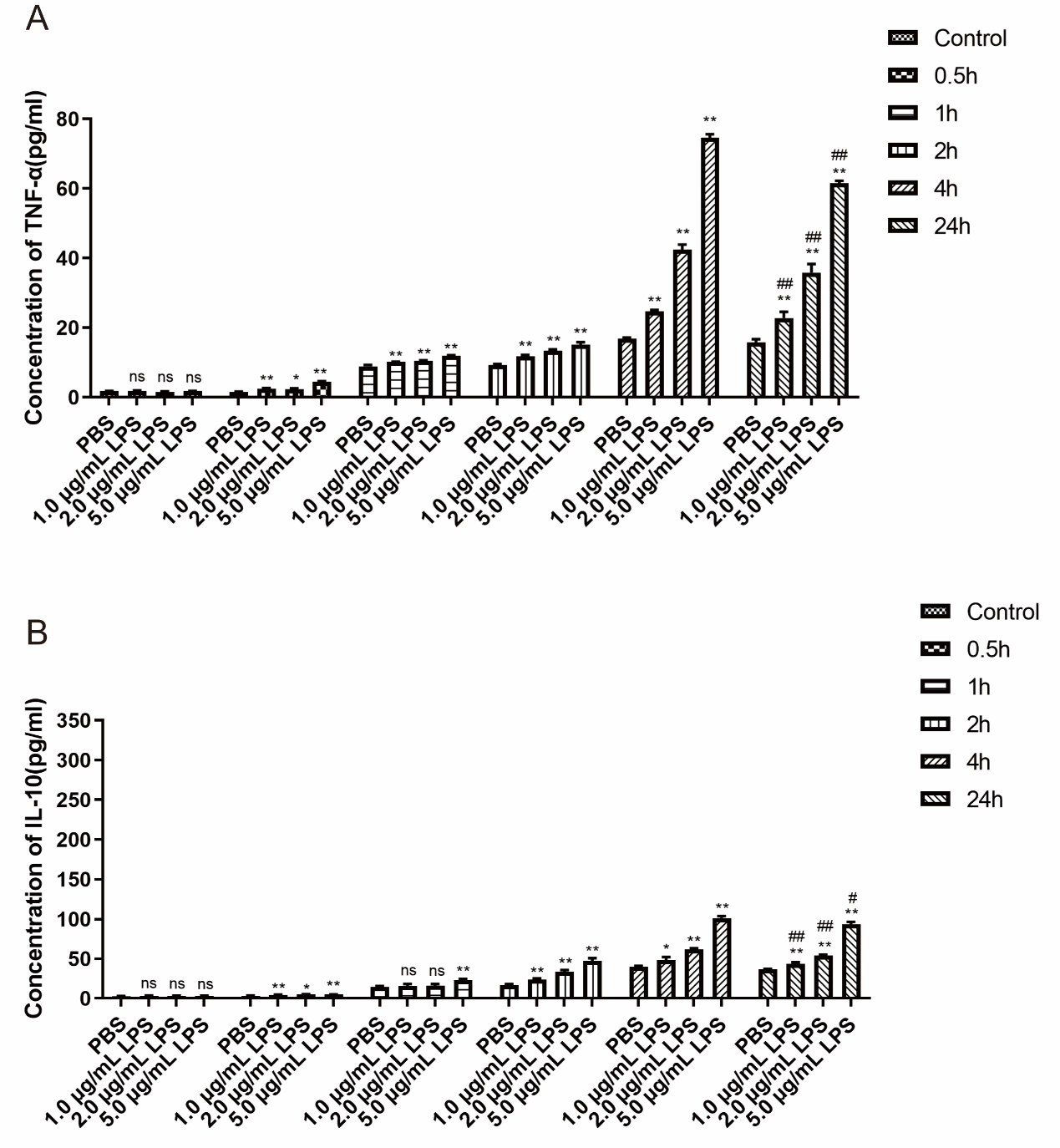


**Supplementary Figure S3.** The establishment of LPS-induced peritoneal macrophages inflammation model. (A-B) peritoneal macrophages were treated with various concentrations of LPS for various time periods. Tumor necrosis factor‐alpha (TNF‐α)and interleukin‐10 (IL‐10)were detected by enzyme-linked immunosorbent assay (ELISA). The data are presented as mean ± SD of at least three independent experiments. *P < 0.05 and **P < 0.01 compared with PBS treatment. #P < 0.05 and ##P < 0.01 compared with LPS treatment in 5.0 μg/mL LPS at 4h. Statistical significance was determined by a Student's t‐test.

.
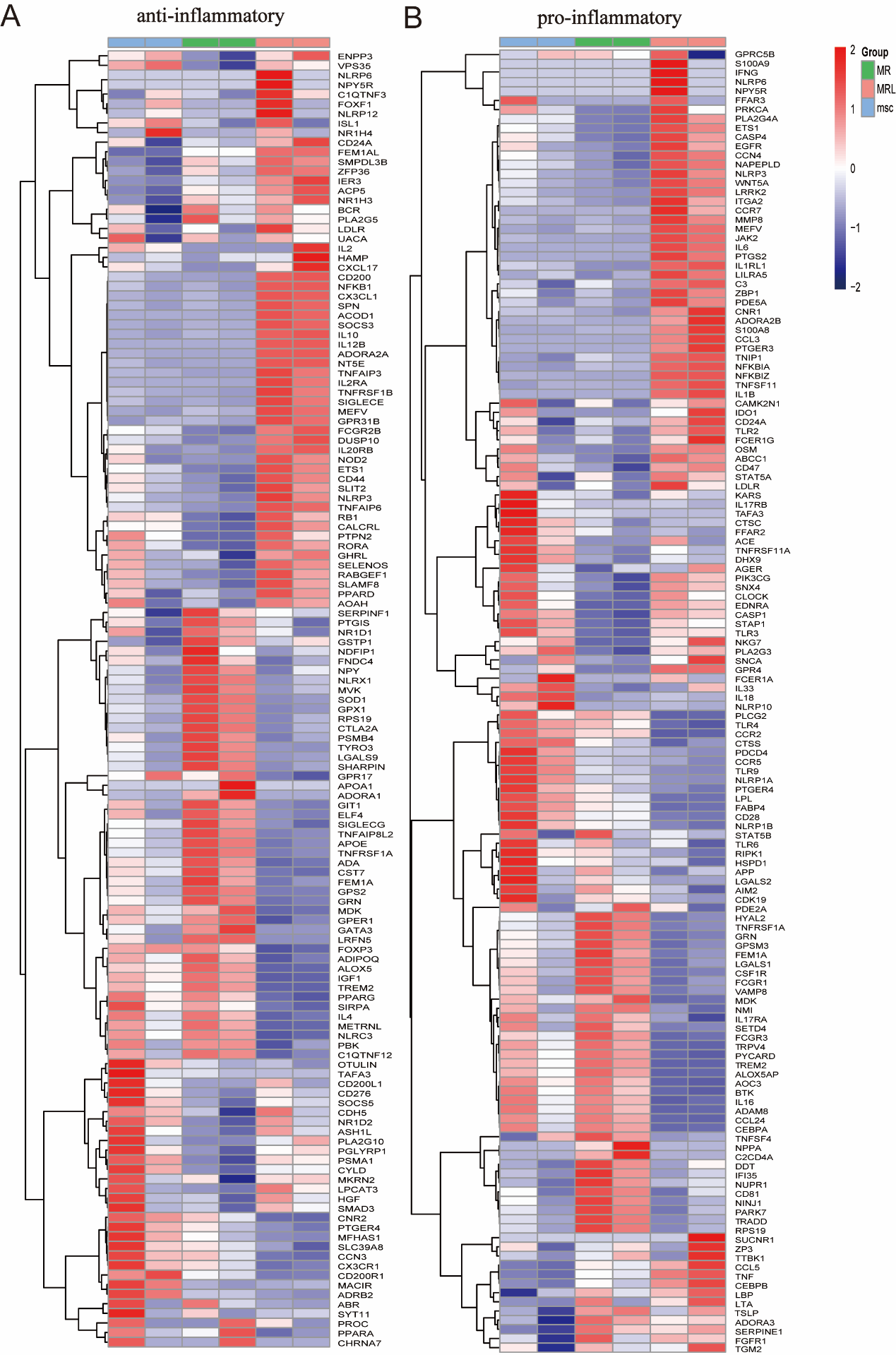


**Supplementary Figure S4.** RNA-seq analysis of MSCs cultured alone or in the presence of untreated or LPS-treated macrophages. (A) Heatmap showing the core genes enriched in Anti-inflammatory pathway. (B) Heatmap showing the core genes enriched in Pro-inflammatory pathway. The abscissa represents the sample number and the ordinate represents the differentially expressed genes. downregulated in blue, upregulated in red.


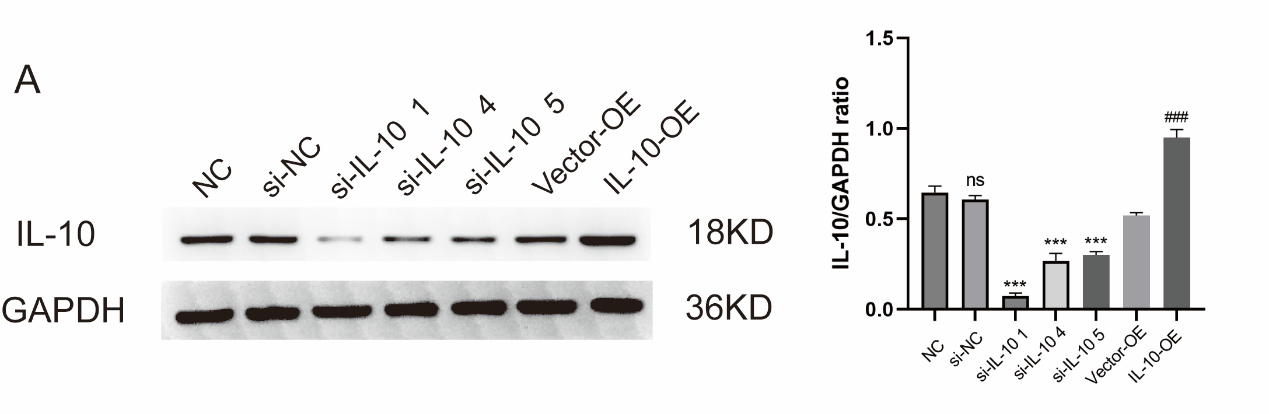


**Supplementary Figure S5.** Overexpression of IL-10 and knockdown via siRNA was assessed by immunoblot analysis. The data are presented as mean ± SD of at least three independent experiments. **P < 0.01 and ***P < 0.001 compared with si-NC group. ###P < 0.001 compared with Vector-OE group. Statistical significance was determined by a Student's t‐test.NC, negative control; OE, overexpression; ns, no significant.
